# Transcriptional Profiling of Host Cell Responses to Virulent *Haemophilus parasuis*: New Insights into Pathogenesis

**DOI:** 10.3390/ijms19051320

**Published:** 2018-04-29

**Authors:** Shulin Fu, Jing Guo, Ruizhi Li, Yinsheng Qiu, Chun Ye, Yu Liu, Zhongyuan Wu, Ling Guo, Yongqing Hou, Chien-An Andy Hu

**Affiliations:** 1 Hubei Key Laboratory of Animal Nutrition and Feed Science, Wuhan Polytechnic University, Wuhan 430023, China; fushulin2016@126.com (S.F.); 1604150051gj@gmail.com (J.G.); 459657811lrz@gmail.com (R.L.); yechun0226@163.com (C.Y.); lyywfy@foxmail.com (Y.L.); zhongywu@163.com (Z.W.); guoling1101@163.com (L.G.); houyq@aliyun.com (Y.H.); AHU@salud.unm.edu (C.-A.A.H.); 2 Hubei Collaborative Innovation Center for Animal Nutrition and Feed Safety, Wuhan 430023, China; 3 Biochemistry and Molecular Biology, University of New Mexico School of Medicine, Albuquerque, NM 87131, USA

**Keywords:** *Haemophilus parasuis*, porcine aortic vascular endothelial cells, vascular damage, infection

## Abstract

*Haemophilus parasuis* is the causative agent of Glässer’s disease in pigs. *H. parasuis* can cause vascular damage, although the mechanism remains unclear. In this study, we investigated the host cell responses involved in the molecular pathway interactions in porcine aortic vascular endothelial cells (PAVECs) induced by *H. parasuis* using RNA-Seq. The transcriptome results showed that when PAVECs were infected with *H. parasuis* for 24 h, 281 differentially expressed genes (DEGs) were identified; of which, 236 were upregulated and 45 downregulated. The 281 DEGs were involved in 136 KEGG signaling pathways that were organismal systems, environmental information processing, metabolism, cellular processes, and genetic information processing. The main pathways were the Rap1, FoxO, and PI3K/Akt signaling pathways, and the overexpressed genes were determined and verified by quantitative reverse transcription polymerase chain reaction. In addition, 252 genes were clustered into biological processes, molecular processes, and cellular components. Our study provides new insights for understanding the interaction between bacterial and host cells, and analyzed, in detail, the possible mechanisms that lead to vascular damage induced by *H. parasuis*. This may lead to development of novel therapeutic targets to control *H. parasuis* infection.

## 1. Introduction

*Haemophilus parasuis* is a small, Gram-negative nicotinamide adenine dinucleotide (NAD)-dependent bacterium that is a member of the family Pasteurellaceae. It is the causative agent of Glässer’s disease in pigs, and can cause huge economic losses in pig production [1]. The typical characteristics of Glässer’s disease are polyserositis, meningitis, and arthritis [2]. So far, 15 serovars of *H. parasuis* have been identified, but >20% of isolates have not been isolated yet [3,4]. The serovar is thought to be an important virulence marker in *H. parasuis* [5]. Serovar 5 of *H. parasuis* is considered to be highly virulent and serovar 4 moderately virulent [6]. Therefore, as one of the most important bacterial respiratory pathogens in pigs, controlling infection caused by *H. parasuis* is crucial.

To date, the pathogenesis of *H. parasuis* has not been resolved in detail. Some virulence factors have been found and confirmed. Previous reports have shown that the *H. parasuis* two-component signal transduction system, CpxRA, confers tolerance to stress and bactericidal antibiotics [7]. Deletion of *vacJ* gene contributes to a decreased survival ratio, complement killing, decreased adhesion to and invasion of PK-15 cells, and reduced biofilm formation [8]. The Δ*arcA* or Δ*cheY* deletion mutant of EP3 strain has reduced virulence and produces less biofilm mass [9,10]. The *htrA* mutant showed significantly attenuated virulence in a murine model of infection [11]. The Δ*rfaE* mutant strain has reduced adherence and invasion of porcine umbilicus vein endothelial cells and PK-15 cells [12]. In addition, the *capD* gene is a novel pathogenicity-associated determinant and is involved in serum resistance of *H. parasuis* [13]. *H. parasuis* causes vascular inflammation and damage, but the mechanism has not been established. Although virulence-related factors may play important roles in the infection process of *H. parasuis* during bacterial invasion and adhesion, pathogenesis infection is a complex process, and the mechanism of vascular damage needs to be studied further.

Bacteria adhere to or invade host cells, resulting in changes in cellular function. Bacteria may initiate interaction with multiple cellular components of target cells and influence host cell gene transcription and expression [14]. Therefore, the initial and subsequent host cell responses to the bacteria are important when trying to comprehend the interaction between bacterial and host cells, which could be determined at the transcriptional level. We can now utilize the RNA sequencing (RNA-seq) method to try and understand the effect of host cell transcription changes induced by *H. parasuis* at the whole genome scale. RNA-seq analysis is a useful and powerful tool that can quickly identify batches of genetic determinants [15]. RNA-seq can rapidly provide unique insights into the processes of bacterial infection and host responses [16,17,18]. In addition, RNA-seq could observe virulence-related factors [19], transcribed intronic and intergenic regions [20], differential isoform expression [21], and alternative splicing [22]. Thus, we used RNA-seq analysis to help us to study the mechanism of vascular damage triggered by *H. parasuis*.

In this study, we investigated the transcriptome of porcine aortic vascular endothelial cells (PAVECs) infected with a highly virulent isolate of *H. parasuis*, to analyze the host cell responses during infection. Our findings will help us to understand better the mechanism of vascular damage induced by *H. parasuis*, which could provide new targets to control and prevent infection.

## 2. Results

### 2.1. Transcriptome Sequencing and Annotation

To evaluate the global picture of host cell transcriptomic response to *H. parasuis* infection and to understand which host factors were involved in the infection and inflammatory immune response, we used RNA-seq on the Illumina platform with cDNA libraries of PAVECs infected or mock-infected with *H. parasuis*. A total of 108,360,513.3 ± 8,018,440.6 raw reads with a Q20 value of 97.7 ± 0.1 aligned to *Sus scrofa* genome were detected in the infected cells, compared with 71,624,665.3 ± 2,420,491.4 raw reads with a Q20 value of 97.9 ± 0.1 in the control cells (Table S1). In addition, 22,047,651 ± 15,438,811 and 40,763,421 ± 660,539 of uniquely mapped reads were obtained from infected and control cells, respectively. The uniquely mapped ratios of 88.5 ± 0.9 and 90.2 ± 0.3 were determined after filtering adapters and trimming ambiguous results. The results suggested that the high quality of these sequencing data can be used for additional analysis.

### 2.2. Identification of Differentially Expressed Genes (DEGs)

**Figure 1:**
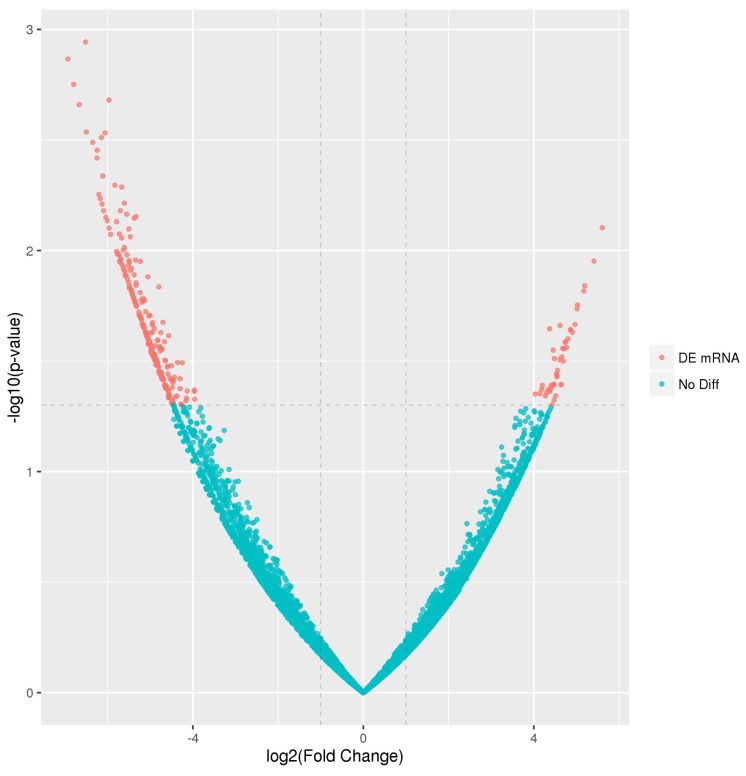
Volcano plot of the differentially expressed genes; (**red**) differentially expressed genes; (**blue**) non-differentially expressed genes.

PAVECs infected with *H. parasuis* showed a greater degree of differential expression. Differential analysis of the transcript expression profiles demonstrated that 281 genes were significantly altered (≥2-fold change, *p* < 0.05); of which 236 were upregulated and 45 downregulated (Figure 1, Table S2).

### 2.3. Functional Annotation of PAVEC Gene Expression Signature Triggered by H. parasuis Infection

**Figure 2:**
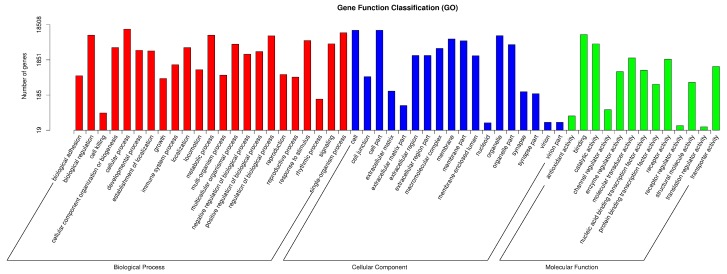
GO functional categories analysis. The *x*-axis displays the categories, and the *y*-axis displays the number of genes in the categories.

To study the host cell response to *H. parasuis* infection, the 281 DEGs were characterized by determining their enrichment analysis using the GO classification and KEGG pathway as the bioinformatics tools. Two hundred and fifty-two genes (89.7%) were distributed to GO categories. Further analysis revealed that they belonged to three types, which were biological process, molecular function, and cellular component (Figure 2). In the biological process group, metabolic process, biological regulation, and signal transduction were the most abundant categories, and other categories were transport process and immune system process. In the molecular process, the most abundant categories were binding, transporter activity, and regulator function.

**Figure 3:**
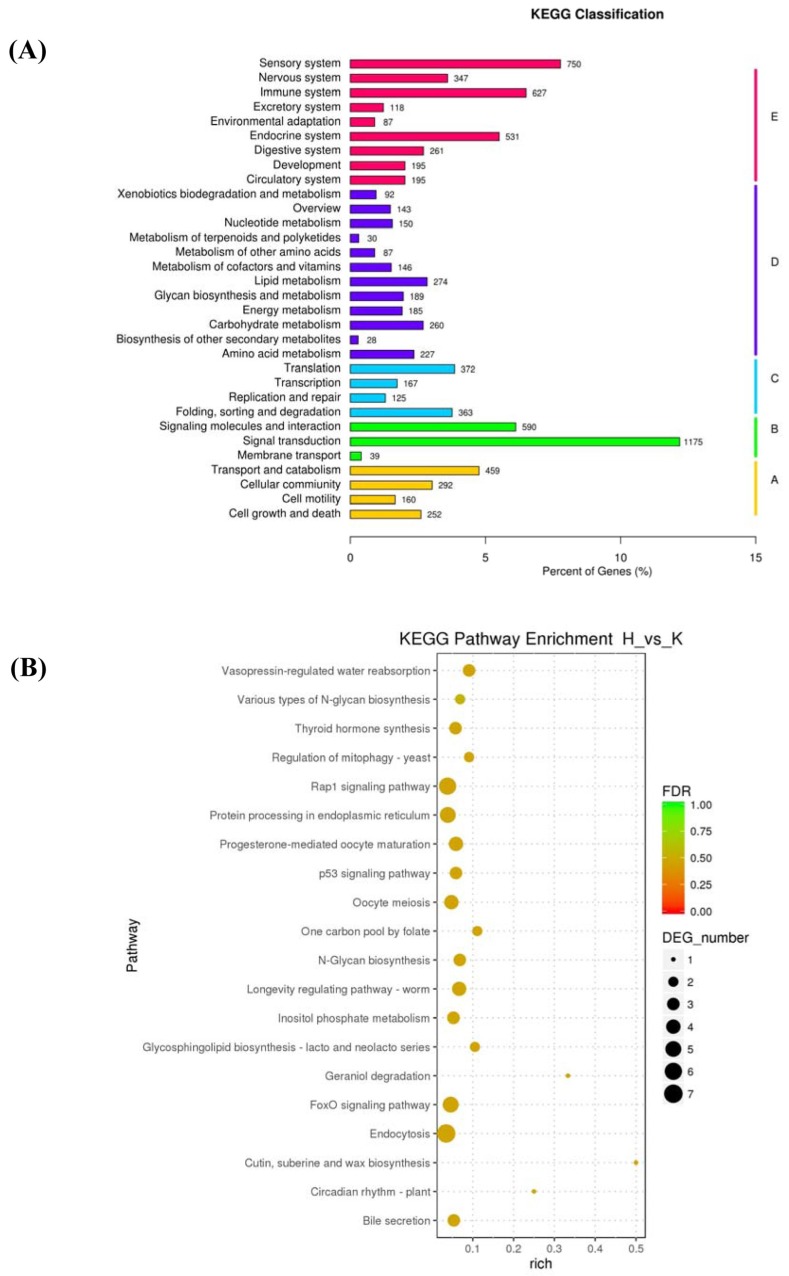
KEGG pathway classification analysis (**A**) and the differentially expressed genes (DEGs) involved in the main pathway (**B**). H: *H. parasuis*. K: the control.

KEGG analysis showed that the DEGs were mainly involved in the signal transduction, sensor system, and immune system, and others participated in the metabolism and cell components (Figure 3A). Furthermore, DEGs involved in the main metabolic and signaling pathways were also investigated. The main metabolic pathways and signaling pathways were Rap1 signaling pathway, endocytosis, FoxO signaling pathway, protein processing in endoplasmic reticulum, progesterone-mediated oocyte maturation, cAMP signaling pathway, PI3K/Akt signaling pathway, Toll-like receptor signaling pathway, and apoptosis, and other regulating pathways (Figure 3B).

**Figure 4:**
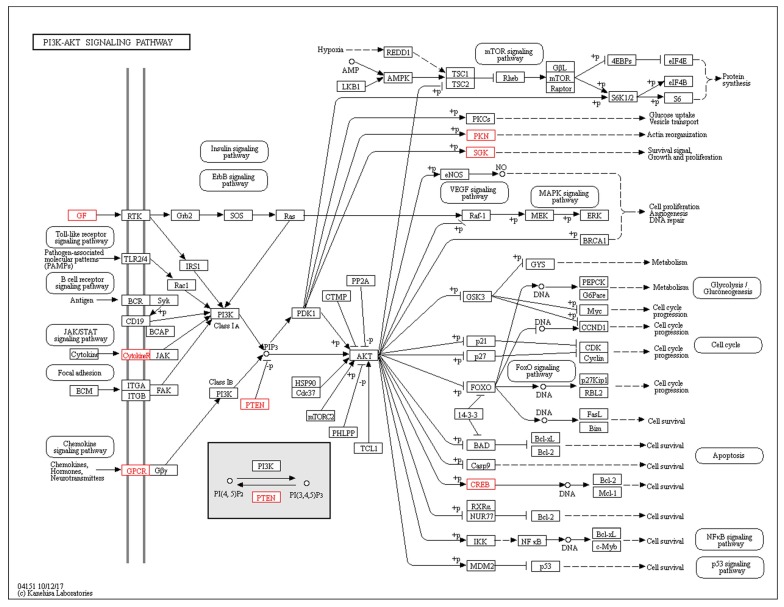
KEGG analysis of the PI3K-AKT signaling pathway. The red genes were upregulated.

DEGs were analyzed with KEGG to predict and study the functions and networks of the encoded proteins. DEGs that were involved in the main and most interesting pathway, and possibly related to vascular damage, were chosen for KEGG analysis using the *Sus scrofa* database. The network of predicted associations for the chosen DEGs in the same pathway is shown in Figure 4. Among the DEGs, seven (ATF6B, PKN2, PTEN, IL7R, F2R, SGK1, and FGF16) were upregulated and located in the PI3K/Akt signaling pathway, which was the one that most of the DEGs participated in (Figure 4). One upregulated DEG, ENSSSCG00000000175, participated in the vascular smooth muscle contraction pathway. In these DEGs, most protein molecules were the critical molecules which were related to each other. Activation of these signaling pathways may be important factors leading to the vascular damage caused by *H. parasuis*.

### 2.4. Analysis of the Association among DEGs of the Main Pathways Using the Search Tool for the Retrieval of Interacting Genes/Proteins (STRING)

**Figure 5:**
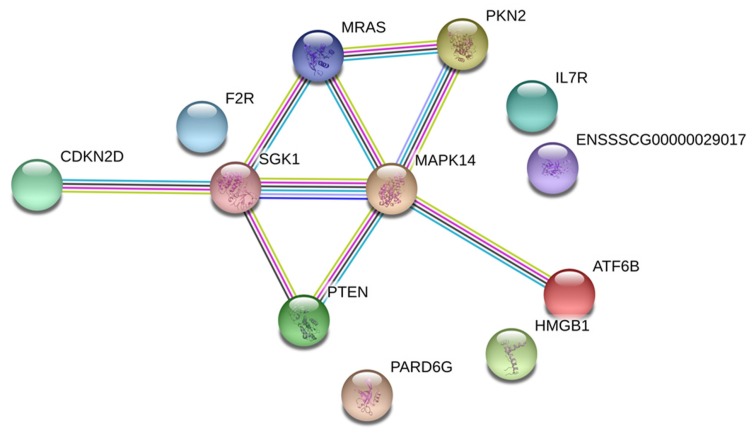
STRING analysis of the relationship between 12 chosen DEGs. The associations between the proteins and the proteins encoded by 12 chosen DEGs were determined using STRING.

DEGs were analyzed by using STRING for predicting the interaction network between the proteins and the proteins encoded by DEGs. The network of the 12 DEGs that participated in the main pathways was constructed using the STRING v10 database to demonstrate the complex associations between those genes. Most of the DEGs were closely associated with each other and displayed a coordinated interactive network, while some proteins were not linked to each other (Figure 5). The network interaction analysis between proteins and proteins speculated that the crosstalk of the DEGs that were chosen coordinately induced vascular inflammation and damage after *H. parasuis* infection.

### 2.5. Real-Time Polymerase Chain Reaction (PCR) Verification of DEGs

**Figure 6:**
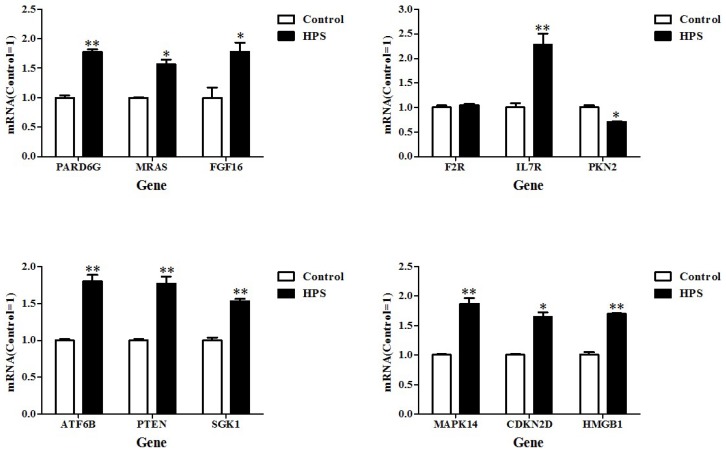
Relative quantification of DEGs for verification by RT-PCR. RT-PCR relative expression levels of selected genes were chosen for the cells infected for 24 h. HPS: *H. parasuis*. * *p* < 0.05; ** *p* < 0.01.

Twelve from the five functional categories were chosen for verification of the DEG data of the RNA-seq using real-time quantitative reverse transcription PCR (qRT-PCR). The gene expression with real-time qRT-PCR was also compared with the data of RNA-seq. Among the chosen 12 genes, 9 genes (PARD6G, MRAS, FGF16, IL7R, ATF6B, PTEN, SGK1, CDKN2D) displayed similar expression levels when compared with the RNA-seq data (Figure 6). Another gene (F2R) did not show obvious changes in expression levels based on the real-time qRT-PCR method. In addition, 10 of the verified DEGs were upregulated and only one was downregulated.

## 3. Discussion

We investigated the global transcriptomic profile of differential gene expression in PAVECs following exposure to *H. parasuis* over 24 h. Consistent with previous research [23,24], our study provided much data that confirmed the vigorous responses of host cells to *H. parasuis*, and this is the first study analyzing the whole transcriptome of PAVECs after *H. parasuis* infection. The results provide new insights into the pathological characteristics of vascular damage caused by *H. parasuis*. We used the RNA-seq approach for global assessment of molecular expression triggered by *H. parasuis* through evaluation of pathway activity changes in vascular inflammation.

Recent studies have reported that the PI3K/Akt signaling pathway is involved in cell proliferation, invasion, and migration [25,26]. Aberrant PI3K/Akt signaling pathway transfer may cause potential defects in some diseases. HMGN5 can regulate expression of PI3Kp85α and p-Akt, and results in tumor cells having malignant potential [27]. Knockdown of HMGN5 could increase the chemosensitivity of human urothelial bladder cancer cells to cisplatin by targeting PI3K/Akt signaling [28]. miR-1268b increases breast cancer cell chemosensitivity via modulation of the PI3K/Akt pathway by targeting ERBB2 [29]. The PI3K/Akt pathway has also become an important contributor to cardiovascular disease due to its role in cardiac growth, angiogenesis, and cardiac hypertrophy [30]. Previous research has demonstrated that vascular endothelial growth factor receptor 1 contributes to *Escherichia coli* K1 invasion of human brain microvascular endothelial cells via recruitment of the PI3K/Akt signaling pathway [31]. Lipopolysaccharide stimulation of vascular smooth muscle cells could induce activation of the PI3K/Akt pathway, and hence, the NF-κB pathway [32]. *Helicobacter pylori* can promote an inflammatory response during infection, which is regulated through c-Met/PI3K/Akt/mTOR signaling pathway activation [33]. Aloperine regulates the inflammatory response in colitis through inhibiting the PI3K/Akt/mTOR signaling pathway in a protein phosphatase 2A-dependent manner [34]. Our results showed that up to seven genes that were upregulated participated in the PI3K/Akt signaling pathway. Among those seven genes, PTEN is important in the PI3K/Akt signaling pathway. PTEN is considered to be a negative regulator of the PI3K/PTEN/Akt signaling pathway, and can regulate cellular functions [35]. PTEN gene induces cell invasion and migration, which have a pivotal effect during the progression of gastric cancer [36]. In light of the important function of PTEN, in the future, it may be developed as a biomarker during *H. parasuis* infection, and targeting it could provide an efficient strategy for therapeutic intervention in vascular disease evoked by *H. parasuis*.

Rap1 is a small GTPase and belongs to the Ras family, which is involved in several cellular signal transduction pathways. Rap1 is necessary for intracellular bacterial pathogen replication-permissive vacuole formation [37]. The enterotoxigenic *E. coli* (ETEC) heat-labile enterotoxin-induced nuclear factor (NF)-κB activation depends upon the cAMP-dependent activation of the Ras-like GTPase Rap1 promoting ETEC adherence to intestinal epithelial cells [38]. *Streptococcus pneumoniae* caseinolytic protease L modulates adherence to A549 human lung cells by inducing and activating Rap1 [39]. Rap1 promotes the production of cytokines via activation of NF-κB in macrophages that prefer a proinflammatory environment that results in atherosclerosis development and progression [40]. In our study, Rap1 signaling pathway was significantly activated in the PAVECs infected by *H. parasuis*, and five genes, GF, GPCR, MRAS, AC, and PAR6 were upregulated. Previous research has shown that PAR6 is involved in cancer initiation, progression and metastasis [41], and blocking the interaction between PAR6 and PKC1 could suppress the growth of lung tumor [42] and human lung cancer cells [43]. Thus, targeted therapies against PAR6 may provide an effective strategy in cancer treatment [44]. Therefore, we speculate that PAR6 participates in the cellular damage process during *H. parasuis* infection, and may serve as a biomarker of pathogenesis of *H. parasuis* infection, and thus, we aim to develop PAR6 as a target to control *H. parasuis* infection.

Our data showed that the FoxO signaling pathway was involved in the process of bacterial infection of PAVECs. Previous research has demonstrated that forkhead box O (FOXO) transcription factors act as tumor suppressors in a wide range of cancers, and are negatively adjusted by the PI3K/PKB/Akt signaling pathway [45]. FoxO transcription factors suppress cancer cell growth and survival [46]. Other studies have shown that FoxO transcription factors enhance tumor development and progression through maintaining cell homeostasis, boosting metastasis, and evoking therapy resistance [47]. The activation of FoxO may be a potential method to postpone aging and reduce onset of Alzheimer’s disease [48] and serve as therapeutic targets in cancer [49]. Thus, we suggest that, in accordance with the tumor suppressive effect of FoxO transcription factors, FoxO activation may have a beneficial regulatory effect on environmental stress and protect against onset of vascular damage induced by *H. parasuis*. The mechanism of this effect needs further study.

In conclusion, our study provides new insights into the host cell responses that are involved in the molecular signaling pathway interactions between bacteria and host PAVECs, using transcriptome profiling. In addition, we analyzed, in detail, the possible mechanisms that lead to vascular damage induced by *H. parasuis*, which may lead to development of novel therapeutic targets to control infection.

## 4. Materials and Methods

### 4.1. Bacteria and Cells

*H. parasuis* SH0165 strain, serovar 5, is a highly virulent strain, which was isolated from the lungs of a commercial pig with arthritis, fibrinous polyserositis, hemorrhagic pneumonia, and meningitis [50,51]. The SH0165 strain was grown in tryptic soy broth (Difco Laboratories, Detroit, MI, USA) or tryptic soy agar (Difco Laboratories) supplemented with 10% newborn calf serum (Sijiqing, Hangzhou, China) and 10 μg/mL NAD (Sigma, Saint Louis, MO, USA) at 37 °C.

This study was conducted in strict accordance with the recommendations of the China Regulations for the Administration of Affairs Concerning Experimental Animals 1988 and Hubei Regulations for the Administration of Affairs Concerning Experimental Animals 2005. The protocol was carried out by China Hubei Province Science and Technology Department (permit number SYXK(ER) 2010-0029). All experimental animals were killed at the end of the experiments.

Three 30-day-old naturally farrowed, early-weaned piglets (Duroc × Landrace × large white), that were confirmed as negative for antibody against *H. parasuis* by INGEZIM Haemophilus 11.HPS.K1 (INGEZIM, Madrid, Spain), and weighing 6–8 kg, were obtained from Wuhan COFCO Meat Product Co., Ltd. (Wuhan, China).

PAVECs were isolated, identified, and cultured as described previously, with minor modifications [52,53]. PAVECs were obtained in small sheets after treatment of aortic lumen (30 min, 37 °C) with 0.1% type I collagenase (Sigma, St. Louis, MO, USA) in M-199 medium (Gibco, New York, NY, USA) containing penicillin–streptomycin solution (Gibco). The suspension was centrifuged at 100× *g* for 15 min, and the PAVECs were resuspended in 5 mL M-199 medium containing 10% fetal bovine serum (Gibco, Victoria, Australia), and cultured in a T-25 tissue-culture plate (Costar, New York, NY, USA). PAVECs were counted and the viability of the cells was detected by trypan blue exclusion.

### 4.2. Bacterial Infection

PAVECs (1 × 10^6^) were seeded into 24-well plates and infected with *H. parasuis* SH0165 strain with a multiplicity of infection of 1. After co-culture for 12 h at 37 °C with 5% CO_2_, the supernatant was removed. The cells were collected and washed three times with 1% sterile phosphate-buffered saline for transcriptomic analysis. The control cells were infected with M-199 medium and treated in the same way. Three individual replicates were designed for the infection experiment.

### 4.3. Construction of Library and Illumina Sequencing

RNA-seq sample preparation and library construction were performed at Shanghai Personal Biotechnology Co., Ltd. The RNA-seq was repeated three times. The total RNA of PAVECs was extracted using TRIzol Reagent (ThermoFisher Scientific, New York, NY, USA) from infected and uninfected cells. Total RNA was purified using the Dynabeads mRNA Purification Kit (ThermoFisher Scientific).

The cDNA libraries were constructed with RNA isolated from PAVECs using the TruSeq Stranded mRNA library preparation kit (Illumina, San Diego, CA, USA) [54]. To obtain mRNA, the poly(A) section was carried out by utilizing RNA purification beads on 0.1–4 μg total RNA of each sample. To synthesize first-strand cDNA, purified mRNA at 10–400 ng was fragmented, and reverse transcriptase and random hexamer primers were utilized. Synthesis of second-strand cDNA was carried out using deoxynucleoside triphosphates containing DNA polymerase, dUTP, and RNase. End repair was achieved by adding dATP to all free 3′ ends, and unique index sequences of the fragments were added with adaptor ligation. The products were amplified by PCR. The quantity and quality of the cDNA libraries were evaluated using an Agilent 2100 bioanalyzer and ABI StepOnePlus Real-Time PCR system. The obtained libraries were sequenced using the Illumina HiSeq 4000 platform. Meanwhile the raw data of RNA-Seq files have been uploaded in NCBI (https://www.ncbi.nlm.nih.gov/geo/) with the accession number GSE113252.

### 4.4. Analysis of RNA-Seq Data

RNA-seq data were analyzed as described previously [55]. The raw data of RNA-seq analysis were filtered by utilizing FASTQC software. The filtered reads were aligned and mapped to the reference genome (Sus-scrofa.Sscrofa10.2.dna.toplevel.fa) by using Tophat2 (http://tophat.cbcb.umd.edu/). To determine gene expression abundance, the unique mapped gene reads were standardized by reads per kilobases per million reads. DEGs were screened using DESeq version 1.18.0 with a fold change of >2 and *p* < 0.05, which were considered as significantly differentially expressed.

The biological pathways involved in differential gene expression were analyzed using DAVID (Database for Annotation, Visualization, and Integrated Discovery), version 6.7. GO and pathway enrichment analysis were determined using DAVID to obtain a list of DEGs [56]. KEGG pathways were identified that were thought to be significantly enriched in the altered genes (*p* < 0.05). The associations between the proteins and the proteins encoded by DEGs were determined using STRING [57].

### 4.5. Validation by Real-Time qRT-PCR

Cell RNA was extracted, and cDNA synthesis was performed by PrimeScript™ II 1st Strand cDNA Synthesis Kit (TaKaRa, Dalian, China). mRNA expression levels of 12 genes were determined by using SYBR Premix Ex Taq (TaKaRa, Dalian, China) in an ABI 7500 real-time PCR system (Applied Biosystems, Foster City, CA, USA). These genes were chosen based on changes in expression level in the infected cells compared with the control cells. Therefore, 10 upregulated and 2 downregulated genes were chosen for validation of mRNA expression levels. The method of primer design was performed as previously reported [58,59]. The length of the primers was between 20 and 25 bases (Table S3). The thermal cycling conditions were denaturation at 95 °C for 15 s, annealing at 56 °C for 30 s and extension at 72 °C for 30 s. The relative gene expression was determined using the threshold cycle method, and the fold changes were calculated by using the 2^−ΔΔ*C*t^ formula [60].

### 4.6. Statistical Analysis

The experimental data were expressed as mean ± SD. The difference between two groups was analyzed using the two-tailed Student’s *t* test. *p* < 0.05 was considered significant. * *p* < 0.05; and ** *p* < 0.01.

## Supplementary Materials

Supplementary materials can be found at http://www.mdpi.com/1422-0067/19/5/1320/s1.
