# Supplementary material for: Transcriptional Profiling of Host Cell Responses to Virulent Haemophilus parasuis: New Insights into Pathogenesis
Source: Int J Mol Sci. 2018 Apr 29;19(5):1320. doi: 10.3390/ijms19051320 (PMC5983834; doi:10.3390/ijms19051320)
Supplement: Supplementary file 1 [file ijms-19-01320-s001.zip › Supplementary Table 1.pdf]

Supplementary Table S1. Statistical summary analysis of RNA-seq datasets of infection cells and control cells.

Samples	Means and SD of Raw			Means and SD of Mapping		
	Raw reads	Clean reads	Q20 Value	Total Mapped Reads	Uniquely Mapped Reads	Uniquely Mapped ratio
Infection cells	108360513.3 ±8018440.6	107734910 ±7965745.8	97.7 ±0.1	24967595 ±17508433	22047651 ±15438811	88.5 ±0.9
Control cells	71624665.3 ±2420491.4	71063982.7 ±2410841.6	97.9 ±0.1	45220205 ±8624953	40763421 ±7660539	90.2 ±0.3
