# Supplementary material for: Transcriptional Profiling of Host Cell Responses to Virulent Haemophilus parasuis: New Insights into Pathogenesis
Source: Int J Mol Sci. 2018 Apr 29;19(5):1320. doi: 10.3390/ijms19051320 (PMC5983834; doi:10.3390/ijms19051320)
Supplement: Supplementary file 1 [file ijms-19-01320-s001.zip › Supplementary Table 3.pdf]

Supplementary Table S3 Primers for qRT-PCR.

Gene	Nucleotide sequence (5'-3')	
β-actin	Forward	TGCGGGACATCAAGGAGAAG
	Reverse	AGTTGAAGGTGGTCTCGTGG
CDKN2D	Forward	GTTTGTGGCTTGTTGGTGTTGAT
	Reverse	TGCCCTTGGGACTTGTCTGTAG
PTEN	Forward	GAAAATGGAGGTCTGTGTGA
	Reverse	TGGAGAAAAGTATCGGTTGG
IL7R	Forward	CACTTCAGAACGCCAGAGACC
	Reverse	CAGAATGACCATCAGAGCCAC
SGK1	Forward	ATGCCAACCCTTCTCCTCCT
	Reverse	AAAACTGCCCTTCCCAATCA
MRAS	Forward	ATGTCACCACCCCAACCCTA
	Reverse	AGCCCAGTCAATGAGCAAAAC
F2R	Forward	CTGAAGGACATAGAGGACCAA
	Reverse	TAGAGGAAGGCTTACGAGAAA
FGF16	Forward	AATGCCTTCTTCCCTCCGTG
	Reverse	GGTTTCTTTGTTCTGTTGCTCCC
PARD6G	Forward	GCGAAGACGAGGCTAAGTGG
	Reverse	TGTTGGAGATGTGGTGGGTG
PKN2	Forward	ACTCTCCAAACCAGCAGCAC
	Reverse	TTCAAAACCGCACACACATC
ATF6B	Forward	CCAGCCATCAGCCACAACAAG
	Reverse	GGGTCCGAAACAGCATTACACG
HMGB1	Forward	CTATCCATTGGTGATGTTGC
	Reverse	TCCTCCTCTTCCTTCTTTTT
MAPK14	Forward	CTGCTGCTTGTCCTGTTCTCTT
	Reverse	ACATCCACCTGTTCTGTTTCTTC
